# Using deep learning to associate human genes with age-related diseases

**DOI:** 10.1093/bioinformatics/btz887

**Published:** 2019-12-17

**Authors:** Fabio Fabris, Daniel Palmer, Khalid M Salama, João Pedro de Magalhães, Alex A Freitas

**Affiliations:** 1 School of Computing, University of Kent, Canterbury, Kent CT2 7NF, UK; 2 Integrative Genomics of Ageing Group, Institute of Ageing and Chronic Disease, University of Liverpool, Liverpool L7 8TX, UK

## Abstract

**Motivation:**

One way to identify genes possibly associated with ageing is to build a *classification model* (from the machine learning field) capable of classifying genes as associated with multiple age-related diseases. To build this model, we use a pre-compiled list of human genes associated with age-related diseases and apply a novel Deep Neural Network (DNN) method to find associations between gene descriptors (e.g. Gene Ontology terms, protein–protein interaction data and biological pathway information) and age-related diseases.

**Results:**

The novelty of our new DNN method is its modular architecture, which has the capability of combining several sources of biological data to predict which ageing-related diseases a gene is associated with (if any). Our DNN method achieves better predictive performance than standard DNN approaches, a Gradient Boosted Tree classifier (a strong baseline method) and a Logistic Regression classifier. Given the DNN model produced by our method, we use two approaches to identify human genes that are not known to be associated with age-related diseases according to our dataset. First, we investigate genes that are close to other disease-associated genes in a complex multi-dimensional feature space learned by the DNN algorithm. Second, using the class label probabilities output by our DNN approach, we identify genes with a high probability of being associated with age-related diseases according to the model. We provide evidence of these putative associations retrieved from the DNN model with literature support.

**Availability and implementation:**

The source code and datasets can be found at: https://github.com/fabiofabris/Bioinfo2019.

**Supplementary information:**

Supplementary data are available at *Bioinformatics* online.

## 1 Introduction

An increasing number of researchers are focussing on solving the ‘ageing problem’, that is, trying to delay ageing in humans. This goal seems to be more and more plausible in the not so distant future: biologists can already considerably extend the lifespan of several animal species, such as the fruit fly and the mouse (De Magalhães *et al.*, 2017). Also, advances in sequencing and analysis have successfully identified several potentially ageing-related proteins (Tacutu *et al.*, 2018). In addition, some organisms (including a few animals) seem to have negligible and even negative senescence (Jones *et al.*, 2014), which indicates that ageing may not be as inevitable as it was first thought.

Another possible outcome of studying ageing as a whole is to reduce the incidence of many different age-related diseases at the same time. This may be more effective than the current approach of treating one disease at a time and could potentially stop the trend of increasing costs of treating age-related diseases (Goldman *et al.*, 2013). To this end, instead of focussing on a single disease, in this work we focus on predicting whether or not human genes are associated with several age-related diseases at the same time using Deep Neural Network (DNN) methods. For a review of machine learning applied to ageing research in general, see Fabris *et al.* (2017).

The machine learning field went through an explosion of DNN applications in the last few years due to the development of new DNN architectures and algorithms, the availability of powerful and accessible processing hardware and the increasing volume of data available to train the models. The areas of bioinformatics and medicine were no different, DNNs have been applied to tackle several problems in these fields, such as MRI image processing (Angermueller *et al.*, 2016), prediction of non-coding DNA function (Quang and Xie, 2016) and prediction of Gene Ontology terms (Kulmanov *et al.*, 2018).

The contributions of this article are 2-fold: (i) to propose and evaluate a novel DNN architecture (using *Keras/tensorflow*) to predict which age-related diseases (our class labels) are associated with human genes (our instances)—note that a gene can be associated with no ageing-related disease or multiple ageing-related diseases and (ii) based on the output of the model, to suggest some genes for further investigation. Our proposed architecture, called ‘Modular DNN’, integrates several data sources to get the final model’s prediction. We compare the Modular DNN approach with more traditional deep learning architectures, with a Gradient Boosted Tree (BT) classifier (*lightgbm* implementation) and with a traditional Logistic Regression (LR) classifier in terms of predictive power.

The remainder of this article is organized as follows: Section 2 describes how the DNN was constructed and how we compiled our data. Section 3 reports the results of our experiments, including a statistical analysis of the predictive performance of the DNN, BT and LR classifiers. Sections 3.3 and 3.4 presents a list of promising genes for further analysis according to our DNN approach. Finally, Section 4 concludes our work.

## 2 Materials and methods

### 2.1 The proposed deep neural network

**Fig. 1.**
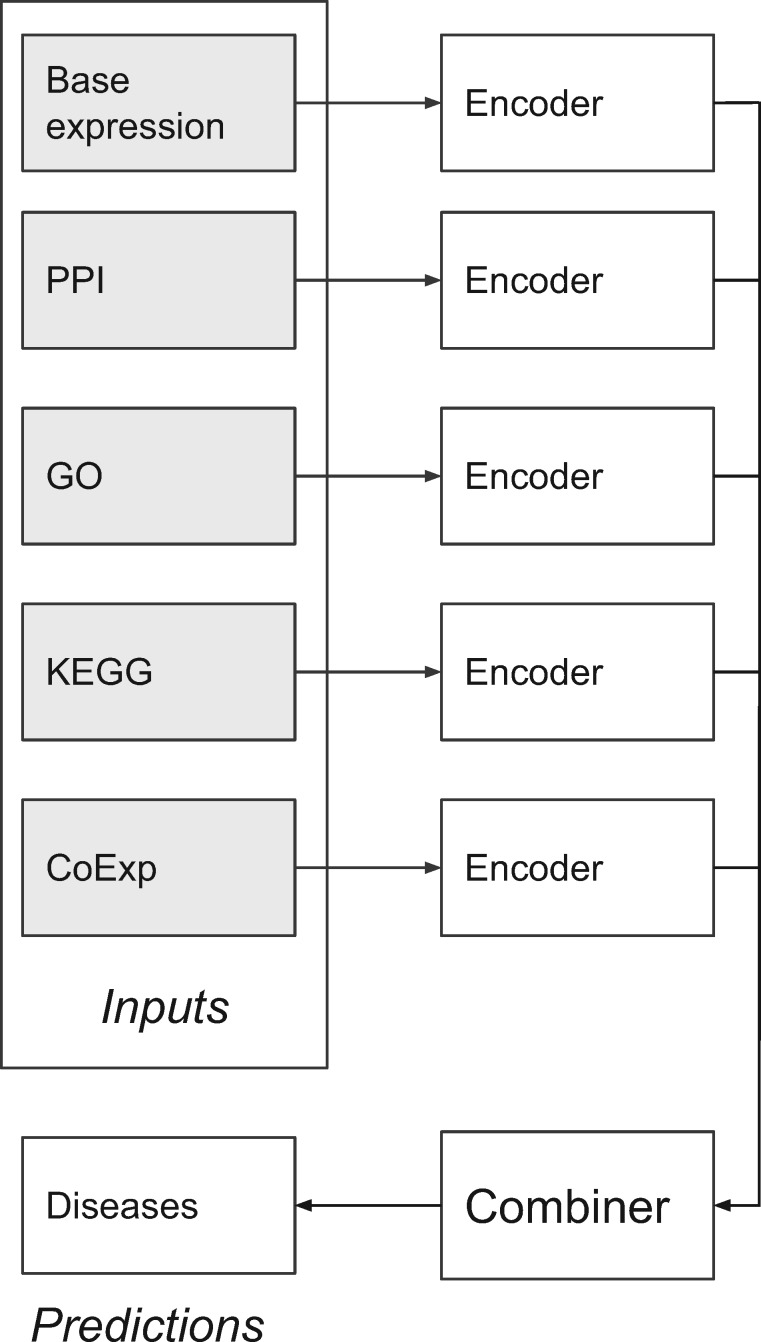
Architecture—Gray nodes represent the inputs coming from several biological databases. Followed by nodes representing the supervised feature extraction modules. The Combiner joins the higher-level features coming from the feature extraction modules to make a final prediction (rightmost node). Each of the encoder nodes, as well as the combiner node, are deep multi-layer neural networks (DNNs)

In this work we investigate a DNN architecture using neurons with *RELU* activation functions and using a stochastic gradient descent algorithm as the optimization engine. Figure 1 shows a high-level graphical representation of the proposed architecture. Our Modular DNN approach comprises several Encoder ‘modules’, one module for each feature type. Each module can be conceptualized as a supervised feature-extraction algorithm specialized in extracting new higher-level features, also referred to as embeddings. Embeddings represent high-dimensional features into dense low-dimensional numerical features.

Each module is trained for a given feature type and is implemented as a DNN with three fully connected layers followed by an output layer that predicts whether or not each instance (gene) is associated with each of the class labels (27 age-related diseases). Each gene may be associated with several class labels at the same time (or none). The hidden layers contain, respectively 64, 32 and 16 neurons. These three hidden layers have a dropout ratio of 0.5 during the training phase. The output layer of an Encoder module contains 27 neurons, one for each of the 27 age-related diseases. The number of trainable weights and neurons in each DNN type is shown in Table 1.

**Table 1.** Number of neurons in the DNN for each type of input feature

Module(s)	No. of trainable weights	No. of input neurons (number of features)	No. of hidden neurons (in all hidden layers)	No. of output neurons
GO	874 491	13 615	112	27
PPI	891 899	13 887	112	27
PathDIP	309 691	4790	112	27
GTex	8507	84	112	27
All (concat.)	2 075 195	32 376	112	27
Modular DNN	2 091 963 (9211 for the Combiner module, 2 082 752 for all four Encoder modules)	32 376	160	27

Note that the predictive performance of the modules can be estimated independently of one another. This will be shown in the next section. After each Encoder module is trained, we remove its output layer (predicting the 27 class labels) and use the outputs of its 16 neurons in the third hidden layer as higher-level features. To combine the individual feature type modules, the outputs of the third hidden layers of all Encoder modules are combined and passed to a final module (the *Combiner*) that makes the final prediction. The Combiner contains two extra hidden layers, the first one containing 32 and the second one containing 16 neurons.

The number of trainable weights of the non-modular DNNs, which do not use the combiner module (rows 1–5 in Table 1) follows the formula $(n_{\text{feats}}+1)\times 64+65\times 32+33\times 16+17\times 27=(n_{\text{feats}}+1)\times 64+3067$, where *n*_feats_ is the number of features in the dataset, shown in the third column of Table 1. Note that the expression 65×32+33×16+17×27 corresponds to the number of neurons in the first hidden layer plus one for the bias (65) times the number of neurons in the second layer (32), followed by the number of neurons in the second layer plus one (33) times the number of neurons in the third layer (16), followed by the number of neurons in the third layer plus one (17) followed by the number of neurons in the output layer (27).

Similarly, the number of *trainable* weights in the combiner module of the modular DNN is (16×4+1)×32+33×16+17×27=9211. The first term of the sum corresponds to the number of projections features extracted from the four modules (16 features from the last hidden layer of each one of the GO, PPI, PathDIP and GTex modules, plus one for the bias) times the number of neurons in the first hidden layer (32), followed by the number of neurons in the first layer plus the bias (33) times the number of neurons in the second hidden layer (16), followed by the number of neurons in the second layer (16) plus the bias times the number of classes (27). Note that this is the *number of trainable weights*, in the Combiner module, after each of the Encoder modules have been trained. The total number of weights is 9211+((13 615+1)×64+65×32+33×16)+((13 887+1)×64+65×32+33×16)+((4790+1)×64+65×32+33×16)+((84+1)×64+65×32+33×16)=2 091 963

Note that each module learns a more compact and richer representation of the input features, which can be more powerful than the original sparse, high-dimensional representation of the features. For this reason, our hypothesis is that combining several specialized modules using a Combiner achieves better predictive performance than the individual modules and than a DNN trained concatenating all feature types into a single input layer. We evaluate this hypothesis in the next section.

We also investigate if sequentially adding modules to the Modular DNN in a forward greedy manner, the Forward Sequential Selection Modular DNN approach (FSS Modular approach), achieves better predictive performance than the modular DNN using all four modules. In this FSS approach the training set is divided into a learning set and an evaluation set, with 2/3 and 1/3 of the training instances, respectively. This FSS approach works by initializing the DNN with no modules, then tentatively testing the performance of the DNN with one module at a time, re-training the Combiner node and measuring the predictive performance of the network on the evaluation set. The best single module is permanently added to the DNN, and the procedure is repeated by tentatively adding one of the three remaining modules at a time to the current DNN and so on. The procedure stops if tentatively adding each of the remaining modules does not increase predictive performance, or of course if all modules were already added to the DNN.

Our modular approach has three advantages when compared to the usual approach of concatenating all features and training a single classifier: (i) the possibility of adding new feature types to the predictive model without the need of re-training the whole model, just the Combiner module; (ii) a more modular, clearer analysis of the effect of each feature type on the predictive performance of the entire DNN, i.e. as feature types are only ‘mixed’ at the Combiner module, the effect of the extracted feature types reaching the Combiner module can be analyzed individually in terms of predictive performance; and (iii) reduced memory consumption (the modules do not need to be loaded into the computer’s memory at the same time).

Our source code, implemented using the *Python* programming language and the *Keras* DNN API, is freely available at https://github.com/fabiofabris/Bioinfo2019

### 2.2 Dataset compilation

Human protein coding genes were downloaded from NCBI BioMart v87 and age-related disease gene associations were obtained from Fernandes *et al.* (2016). These disease associations represent an age-related disease-specific subset of the Genetic Association Database (Becker *et al.*, 2004), a large database of genetic association study results collected until 2014. As such, a gene having a positive class label for a disease association indicates that when this database was frozen in 2014, it contained SNPs associated with the given disease for that gene.

After this list of gene–diseases associations was compiled, we combine each gene with the four feature types used in this work (GO, PPI, PathDIP and GTex). GO term–gene associations were collected from the NCBI website using the following query:

“Homo sapiens”[Organism] AND “source_genomic”[properties]

AND “genetype protein coding”[Properties] AND alive[prop])

PPI data was collected from BioGRID (database version 3.4.146), and PathDIP information was collected from the PathDIP website (version 7). Our datasets are available at https://github.com/fabiofabris/Bioinfo2019.

## 3 Experiments

### 3.1 Comparing the Modular DNN approach with the standard DNN and other methods

In this section, we compare our Modular DNN approach with five baselines: (i) the standard DNN (trained using the individual feature types and the dataset with all concatenated feature types); (ii) the Gradient BT (Ke *et al.*, 2017) algorithm (a state-of-the-art classifier) also using individual feature types, the concatenated dataset and a stacking approach; (iii) the FSS Modular DNN approach; (iv) a ‘Naive’ classifier that ranks the genes in terms of study ‘popularity’—that is, more studied genes are ranked as ‘more associated with the diseases’ than less studied genes. For GO, PPI and PathDIP datasets, we define ‘study popularity’ as the number of terms associated with the gene. If the gene is not present in the dataset at all, the popularity of the gene is ‘0’ by definition. For the GTex feature type (a unbiased feature), we assume that all genes present in the dataset have popularity ‘1’, and the genes not present in the dataset have popularity ‘0’. We also combined the previously described ‘frequency measures’ by adding up all four popularity counts, creating a fifth ‘naive’ classifier that uses information from all feature types. The Naive classifier is a useful heuristic to test if the sophisticated classification algorithms (DNN and BT) are going beyond simply assigning the positive class label to well-studied genes Gillis and Pavlidis (2011). (v) The LR classifier from *Sklearn* using L2 regularization and default parameters.

We have estimated the performance of the classification algorithms using the popular 10-fold cross-validation procedure. This procedure divides the dataset into 10 folds of approximately equal size. Next, each fold is used as a validation dataset and the other nine folds used as the training dataset. The performance of the classification algorithm is estimated by averaging the performance in the 10 validation sets. Note that the predictive performance of each one of the 27 classes is calculated independently using the AUROC measure and averaged to get the final measure of predictive performance for the whole model.

Table 2 shows the predictive accuracy results across feature types and corresponding combination approaches. The first column shows the feature type or combination approach/method being analyzed, with the sixth and seventh rows showing the proposed Modular and FSS Modular DNN approaches. The second column shows the percentage of unknown genes in each feature type—by unknown gene we mean a gene for which the values of all features (of a given type) are unknown, because there is no annotation of that feature type for the gene. For instance, a gene without any GO term annotations is called an ‘unknown’ gene for that feature type. Note that the feature type with the largest proportion of unknown genes is the PPI feature type, with 19.4% unknown genes. The next four columns present the experimental conditions as follows. The third column shows the AUROC for the DNN algorithm when using the class label frequencies in the dataset as the probabilities of predicting the corresponding classes for the unknown genes. The fourth column shows the AUROC results for the DNN algorithm when discarding the unknown genes altogether. The next four columns show equivalent information for the Gradient BT and LR algorithms. The last column shows the results of the Naive approach. Note that the last row shows the result of a stacking approach (Witten *et al.*, 2016) that consists of training a meta-level BT or LR using as features the class probabilities output by the four base level BTs or LRs trained with the individual feature types.

**Table 2.** Comparing AUROC results of our Modular DNN approach (in bold) with the individual feature types and the full dataset concatenating all features (Concat. all feats.) using the DNN, BT and LR algorithms

Feature type or classification method/approach	% of unknown genes in the feature type	AUROC values						
		DNN		BT		LR		Naive
		pred. all genes	pred. known genes only	pred. all genes	pred. known genes only	pred. all genes	pred. known genes only	pred. all genes
GO	4.0%	0.8498	0.8583	0.8520	0.8468	0.7900	0.7981	0.7995
PPI	19.4%	0.6381	0.6897	0.6585	0.6782	0.6844	0.6889	0.6080
PathDIP	16.8%	0.7535	0.8051	0.8392	0.8314	0.7600	0.7607	0.7817
GTex	4.1%	0.7507	0.7476	0.7156	0.7114	0.7173	0.7233	0.5062
Concat. all feats.	0.0%	0.7548	0.7548	0.8794	0.8794	0.8059	0.8059	0.8105
**Mod. approach**	0.0%	**0.8795**	**0.8795**	NA	NA	NA	NA	NA
FSS Mod. approach	0.0%	0.7525	0.7525	NA	NA	NA	NA	NA
Stacking	0.0%	NA	NA	0.7301	0.7301	0.8711	0.8711	NA

*Note*: This table also shows the results when varying the strategy to deal with ‘unknown genes’—i.e. genes for which the values of all features (of a given feature type) are unknown—e.g. a gene where all GO term features have missing values. Columns 3, 5 and 7 show the results when classifying all genes (including unknown genes) while columns 4, 6 and 8 shows the results when ignoring unknown genes. The last column shows the results for the ‘Naive’ approach. For the AUROC results of our approach for the individual diseases, please consult the Supplementary File ‘auroc_per_disease.xlsx’.

First, we will analyze the predictive performance of the DNN classifiers (columns 3 and 4) using four types of experimental conditions: (i) trained using one feature type at a time (rows 1–4), (ii) using a full dataset concatenating all feature types (row 5), (iii) using our Modular DNN approach (row 6) and (iv) using the FSS Modular DNN approach (row 7).

Table 2 shows that our new approach (Modular DNN) achieves better predictive performance than all other individual feature types, and also better performance than the FSS Modular approach and the ‘Concat. all feats.’ approach, which uses a concatenation of all feature types, training a single large, non-Modular DNN. Also, when using the non-Modular DNN trained with a single feature type (rows 1–4), the AUROC calculated using only known genes (fourth column) is greater than the AUROC when using all genes (third column) in three out of four cases. The only exception is the GTex feature type, but the difference is very small (0.0031). This generally greater AUROC when predicting only known genes is expected, as removing validation instances representing unknown genes tends to produce an ‘easier’ classification problem. Note that the Modular DNN approach classifies all genes and still has better predictive performance than the approaches that do not classify all genes (including BT and LR approaches), being clearly superior both in terms predictive power and classification coverage.

By analyzing the learning curves (presented as Supplementary Material), we can clearly see that the DNN models learned from the ‘PPI’ and ‘Concat. all feats.’ datasets are overfitting, i.e. after a certain number of epochs, the error rate in the testing set starts to increase, whilst the error rate keeps decreasing in the training set. This overfitting explains why those feature types had poor predictive performance.

Comparing the results of the Modular DNN and the BT algorithm, overall, the Modular DNN approach (columns 3 and 4, sixth row) achieves higher AUROC values than BT across all individual feature types (columns 5 and 6, rows 1–4). Comparing columns 3 and 5, we can see that for the individual feature types (rows 1–4), BT achieves higher AUROC values when using all genes (including unknown genes) than the DNNs in three out of four feature types (the only exception is the GTex feature type). Also, the performance of BT using stacking (last row) is not competitive in comparison with BT using all features (row 5).

Interestingly, it seems that the BT approach performs worse when the unknown genes are removed from the validation set. Comparing columns 5 and 6, the AUROC of BT *decreased* in three out of four occasions when discarding unknown genes, performing worse than the DNN in three out of four cases in this scenario, although the differences are smaller than 0.01 in all cases. This counter-intuitive result is due to the fact that the positive class (disease) probabilities the BT algorithm is assigning to the known genes are in general higher than the probabilities assigned to the unknown genes (which have a lower disease frequency in the dataset than the known genes). Hence, the unknown instances are correctly ranked below most true positives most of the time, which may ‘inflate’ the AUROC results.

As an example, consider an algorithm that randomly assigns high positive class label (disease) probabilities to the known genes. The AUROC of this classifier would be 0.5 on average. Next, assume that we assign low positive class probabilities to the unknown genes (which have a lower disease frequency in the dataset than the known genes). The final AUROC of this experiment (combining both sets of predictions) would be higher than 0.5, as the positive class ‘density’ of the set of known genes is higher than the positive class ‘density’ of the set of unknown genes.

The LR classifier (columns 7 and 8) had reasonable predictive performance in some feature types, especially when using the PPI feature type predicting all genes, where it outperformed both the DNN and BT algorithms. The best overall performance for the LR algorithm (using the stacking approach) is close to the best DNN result (the Modular Approach).

Finally, the Naive approach had considerably worse performance than the BT classifier across all feature types. The Naive approach outperformed the DNN in two feature types (PathDIP and Concat. all feats.). The Naive approach also outperformed LR when using two feature types (GO and PathDIP). However, the best AUROC achieved by the Naive classifier (0.8105) is considerably inferior to the best AUROC of the Modular Approach (0.8795).

### 3.2 Statistical analysis of the results

In this section, we perform a statistical analysis to compare the performance of the Modular DNN algorithm with other DNN approaches, the Gradient BT and the Linear Regression classifier across several feature types considering the approaches that use unknown genes (third, fifth and seventh columns of Table 2). We do not compare the results of other columns as the underlying datasets are not the same (they have different numbers of instances). Also, to save space, we do not show the results comparing the Modular DNN with the Naive approach since every test resulted in a *P*-value very close to zero, i.e. the Modular approach is clearly statistically significantly superior to the Naive approach.

We use a Bayesian test of statistical significance and a traditional Null Hypothesis Significance Testing (NHST), both based on the Wilcoxon signed rank test. The motivation is to use not only the traditional NHST analysis, but also a more modern Bayesian analysis involves several major drawbacks of the popular NHST analysis, as discussed in Greenland *et al.* (2016), Benavoli *et al.* (2017), Goodman (2008), Stang *et al.* (2010). For each algorithm pair, we use the paired per-fold AUROC to test if they are significantly different. Note that the 10 fold are divided always in the same way, i.e. they contain the same instances across all tests.

Table 3 shows the results comparing the Modular DNN with the other approaches. The first column shows the baseline being compared to the Modular DNN (the traditional DNN, the BT or the LR classifiers), the second column shows the feature type, the third column shows the AUROC values, the fourth column shows results for the Bayesian test of statistical significance and the fifth column shows the *P* values of the NHST, under the null hypothesis of equivalency of the classifiers’ AUROCs.

**Table 3.** Comparing the Modular DNN approach (AUROC = 0.8795) with the DNN, BT and LR approaches using Bayesian hypothesis testing and the traditional NHST

Base.	Feature type or approach	AUROC	Bayesian *P* values (DNN, ROPE, Base.)	NHST *P* values
DNN	GO	0.8498	0.9995, 0.0005, 0.0000	0.0051
	PPI	0.6381	1.0000, 0.0000, 0.0000	0.0051
	PathDIP	0.7535	1.0000, 0.0000, 0.0000	0.0051
	GTex	0.7507	1.0000, 0.0000, 0.0000	0.0051
	Con. all feats.	0.7548	1.0000, 0.0000, 0.0000	0.0051
	FSS Mod. app.	0.7525	0.9989, 0.0011, 0.0000	0.0069
BT	GO	0.8520	0.9980, 0.0020, 0.0000	0.0093
	PPI	0.6585	1.0000, 0.0000, 0.0000	0.0051
	PathDIP	0.8392	0.9999, 0.0001, 0.0000	0.0051
	GTex	0.7156	1.0000, 0.0000, 0.0000	0.0051
	Con. all feats.	0.8794	0.1086, 0.7685, 0.1229	0.8785
	Stacking	0.7301	1.0000, 0.0000, 0.0000	0.0051
LR	GO	0.7900	1.0000, 0.0000, 0.0000	0.0051
	PPI	0.6844	1.0000, 0.0000, 0.0000	0.0051
	PathDIP	0.7600	1.0000, 0.0000, 0.0000	0.0051
	GTex	0.7173	1.0000, 0.0000, 0.0000	0.0051
	Con. all feats.	0.8059	1.0000, 0.0000, 0.0000	0.0051
	Stacking	0.8711	0.3920, 0.5588, 0.0492	0.3863

The three values reported in the fourth column of Table 3 represent, respectively:

1. the probability of the Modular DNN being better than the classifier learned using the feature type or approach in the first column (the baseline classifier);
2. the probability of the AUROC of the two classifiers being in the Region Of Practical Equivalency (ROPE), which means the difference in their AUROC values is less than 1%; and
3. the probability of the baseline classifier being better than the Modular DNN classifier.

Overall, the Modular DNN statistically significantly outperformed (at the significance level α=0.05) the baseline approaches in every test with two exceptions: (i) when using the Gradient BT and the ‘Concat. all feats.’ dataset, the probability of the Modular DNN being better than the BT algorithm was 0.1086 according to the Bayesian test, and the null hypothesis could not be rejected. We could still say that the Modular DNN is *at least* equivalent to the BT approach with *P *=* *0.8771 (adding up the *P* values for the DNN and ROPE in the 11th row of Table 3); and (ii) when using the LR model and the stacking approach, the probability of the Modular DNN being better than LR was 0.3920 according to the Bayesian test. Note, however, that the probability that the LR algorithm is better than our DNN approach is low (0.0492).

## 3.3 Analyzing the output of the Modular DNN

In the last step of our analysis, we mapped our instances (genes) into a 16D space by training our Modular DNN using all available instances and retrieving the 16-dimensional projection of the training instance from the values output by the 16 neurons of the last hidden layer of the Combiner module. This dense 16D projection is known as an *embedding*. Next, for each class (disease) to be predicted, we multiply each element of the embedding vector by its corresponding weight in the network edge leading to the output layer’s node representing that class in the Combiner module. This final step creates 27 16D vectors for each instance, one vector per class.

Given these 16D data spaces, for each class (disease), we analyzed the projections of the instances, identifying several clusters of instances (genes) annotated with a positive class label (i.e. a disease). In some cases, instances annotated with the negative class label (i.e. *not* annotated with the corresponding disease) are also located in these clusters. These negative instances are clear targets for further analysis since they are ‘close’ to positive instances and could be wrongly annotated in our data. Recall that negative labels are less reliable [lack of evidence is not the same as evidence for the absence of disease (Fabris *et al.*, 2018)].

To automatically identify these negative instances, we have proceeded as follows. For each one of the 27 classes, we have identified all negative instances that have at least 9 neighbours with the positive class label among its 10 nearest neighbours (NNs) in the original 16D space extracted from the DNN (after multiplying the instance embeddings by the corresponding output weights). Due to the stochastic nature of the DNN training algorithms, we repeated this procedure 30 times (varying the random seed), re-training the Combiner module using a different random seed and reported the candidate genes satisfying the nine neighbours conditions in at least 20 out of the 30 randomized runs. Most classes did not have any gene with a negative class label satisfying the ‘9-positive-neighbours’ condition in at least 20 randomized runs. The condition is satisfied only for the classes ‘Associated with Heart Diseases’ (two candidate genes), ‘Associated with Myocardial Infarction’ (one candidate gene) and ‘Associated with Type 2 Diabetes’ (three candidate genes).

Due to space limitations, we show in Table 4 two out of the six interesting negative instances (genes *TGFB1* and *IL1B*, both neighbours of many genes annotated with the ‘Associated with Type 2 Diabetes’ class label). The other four instances satisfying the 9-positive-neighbours conditions are shown as Supplementary Material. Table 4 consists of two sub-tables, each starting with the identifier of the candidate gene (the gene annotated with the negative class label and having at least nine positive neighbours) and the number of times the gene was selected as a candidate in the 30 randomized runs of the DNN. In the next row we show the average, minimum and maximum positive class label probabilities across the 30 runs of the algorithm. Next, we show a list of the candidate gene’s neighbours annotated with the positive class label, followed by the list of neighbours annotated with the negative class label, both with the number of times the gene was in the NN list of the candidate gene across the randomized experimental runs.

**Table 4.** List of negatively labelled candidate genes with at least nine positive neighbours annotated with the label ‘Associated with Type 2 Diabetes’ appearing in all 30 runs of the Modular DNN

Candidate gene: ***TGFB1* (transforming growth factor beta 1).**	
Found in 30 out of 30 randomized runs.	
Avg. prob.: 0.4211/min. prob.: 0.1257/max. prob.: 0.7044	
Times in NN list	Positive neighbouring genes
30	*APOA1* (apolipoprotein A1)
30	*LEP* (leptin)
30	*IFNG* (interferon gamma)
30	*PTGS2* (prostaglandin-endoperoxide synthase 2)
30	*VEGFA* (vascular endothelial growth factor A)
30	*ADIPOQ* (adiponectin, C1Q and collagen domain containing)
30	*PPARG* (peroxisome proliferator-activated receptor gamma)
30	*APOA4* (apolipoprotein A4)
28	*PPARGC1A* (PPARG coactivator 1 alpha)
2	*CYP1A1* (cytochrome P450 family 1 subfamily A member 1)

Times in NN list	Negative neighbouring genes

30	*IL1B* (interleukin 1 beta)
Candidate gene: ***IL1B* (interleukin 1 beta).**	
Found in 30 out of 30 randomized runs.	
Avg. prob.: 0.4954/min. prob.: 0.1451/max. prob.: 0.8449	

Times in NN list	Positive neighbouring genes

30	*APOA1* (apolipoprotein A1)
30	*LEP* (leptin)
30	*APOA4* (apolipoprotein A4)
30	*AGT* (angiotensinogen)
30	*PTGS2* (prostaglandin-endoperoxide synthase 2)
30	*VEGFA* (vascular endothelial growth factor A)
29	*IFNG* (interferon gamma)
27	*TNF* (tumour necrosis factor)
26	*APOE* (apolipoprotein E)
8	*ADIPOQ* (adiponectin, C1Q and collagen domain containing)

Times in NN list	Negative neighbouring genes

30	*TGFB1* (transforming growth factor beta 1)

*Note*: Each sub-table shows in its heading the name of the candidate gene and the average, minimum and maximum positive class label probabilities across the 30 randomized runs. Next, we show the list of positive neighbours and the list of its negative neighbours (if any) of the candidate gene. The sub-tables also show the number of times the gene was in the NN list of the candidate gene.

Summarizing Table 4, our analysis suggests that the genes *TGFB1* and *IL1B* are good candidates for association with type 2 diabetes. In fact, both *TGFB1* and *IL1B* genes are established to be involved in the pathogenesis of diabetic nephropathy (Chang *et al.*, 2016; Stefanidis *et al.*, 2014). *IL1B* in particular has previously been associated with coronary heart disease in some genetic backgrounds (Rai *et al.*, 2016), which in turn shares many genetic pathways with type 2 diabetes and may share associated genetic variants (Zhao *et al.*, 2017).

## 3.4 Analyzing the probabilities output by the Modular DNN

We present in Table 5 the top five negative genes in terms of average probability of being associated with a positive class label by our Modular DNN algorithm across 30 randomized runs. Due to space limitations, we present only the results for brain diseases, neoplasms and myocardial infarction, which are the most interesting results. The full results are presented as Supplementary Material. We have repeated the experiment 30 times due to the stochastic nature of the DNN training algorithm. Note that negative genes predicted with a relatively high positive class label probability are clear candidates for further analysis.

**Table 5.** List of the top five negative genes in terms of average positive class label probability across 30 randomized runs of the modular DNN and three disease types

Candidate gene	Avg. prob.	Min. prob.	Max. prob.
Brain disease (99% percentile of avg. prob.: 0.0942)			
*AGT* (angiotensinogen)	0.5764	0.2659	0.8631
*LEP* (leptin)	0.4762	0.2315	0.7186
*APOA4* (apolipoprotein A4)	0.4737	0.2214	0.7014
*PTGS2* (prostaglandin-endoperoxide synthase 2)	0.4393	0.2052	0.6542
*TGFB1* (transforming growth factor beta 1)	0.4306	0.1977	0.6367
Neoplasm (99% percentile of avg. prob.: 0.0784)			
*AGT* (angiotensinogen)	0.5128	0.1728	0.8357
*APOA4* (apolipoprotein A4)	0.4228	0.1439	0.6891
*APOA1* (apolipoprotein A1)	0.3610	0.1338	0.5780
*ADIPOQ* (adiponectin, C1Q and collagen domain containing)	0.3507	0.1243	0.5686
*APOB* (apolipoprotein B)	0.2856	0.1036	0.4588
Myocardial infarction (99% percentile of avg. prob.: 0.0808)			
*TNF* (tumour necrosis factor)	0.5283	0.1279	0.8312
*LEP* (leptin)	0.4138	0.1050	0.6374
*IFNG* (interferon gamma)	0.3532	0.0888	0.5545
*PPARGC1A* (PPARG coactivator 1 alpha)	0.3050	0.0734	0.4603
*CYP1A1* (cytochrome P450 family 1 subfamily A member 1)	0.2844	0.0736	0.4284

*Note*: The table shows the class label associated with the gene, the average, minimum and maximum probabilities across the 30 runs.

Table 5 shows three sub-tables, each sub-table shows in its header the class label under consideration and the 99% percentile of the probability that the gene belongs to the positive class (i.e. 99% of the genes have a lower average positive class label probability than the one shown). Note that the probabilities of the genes in the table are much greater than the 99% percentile probability, meaning that these probabilities are much greater than the vast majority of the probabilities output by the classifier. Each sub-table shows in its first column the gene identifier and name. The second, third and fourth columns show, respectively, the average, minimum and maximum probabilities across the 30 randomized runs of the DNN.

There is biological plausibility for the top gene–disease associations for each disease in Table 5: the gene *AGT* (angiotensinogen) is implicated in both Alzheimer’s disease (Mateos *et al.*, 2011) and breast cancer (Herr *et al.*, 2008), with evidence that the renin-angiotensin system of which it is a part may be closely involved in cancer processes (Wegman-Ostrosky *et al.*, 2015) and may even be a target for the treatment of cancer (Vallejo-Ardila *et al.*, 2018). The gene *TNF* (tumour necrosis factor) is expressed in the myocardium in response to mechanical overload or ischaemic injury (Kurrelmeyer *et al.*, 2000), while inhibition therapy of *TNF* may reduce the risk of myocardial infarction in patients with rheumatoid arthritis, a typically high risk group (Low *et al.*, 2017). Interestingly, due to the differences in methodology, neither of these genes were identified by our analysis in the previous section.

## 4 Discussion

Overall, in terms of predictive performance, the Modular DNN approach performed better than using individual feature types and using all features at the same time without the modular architecture (the ‘Concat. all feats.’ approach). Also, the Modular DNN outperformed the Gradient BT approach and the Linear Regression classifier in both the individual, ‘Concat. all feats.’ and ‘stacking’ feature settings.

Also, we presented two ways of extracting useful information from DNN models. First, for each negative instance (human gene), we extracted the neighbouring genes by treating the output of the last hidden layer of the DNN as a mapping to a 16-dimension space. Next, the negative human genes in a neighbourhood with at least 9 out of 10 genes were considered good candidates for further analysis. The second approach identified the negative genes with high positive probability for the disease class labels according to the DNN model, which are also candidates for further analysis.

We conclude that both approaches uncovered genes with biological plausibility of being associated with some age-related diseases. However, both approaches result in different candidate genes, which suggests the complementarity of both approaches and that they should be used in concert while looking for candidate genes for further analysis. As future work, we plan to use these genes in conjunction with the human diseasome (Goh and Choi, 2012) to suggest drugs that target highly ranked candidate genes and also try to identify functional modules in the candidate gene list. We also plan to break down the analysis of the results by gene category, for instance, genes associated with certain biological pathways in all four individual feature types. This may help identify larger groups of genes that are clearly associated with the diseases.

Note that the genes identified in our analysis are highly studied. This is expected, as popular genes tend to have more annotations, and consequently, more opportunity to possess a property that is strongly linked to ageing-related diseases. To attenuate this, we plan as future work to use only feature types (such as levels of gene expression) that are *not* influenced by the ‘study popularity’ of the gene.

In particular, according to our projection approach, genes *TGFB1* and *IL1B* are good candidates for association with type 2 diabetes. Furthermore, according to the approach that investigates negative genes with high positive probability as output by the DNN, the gene *AGT* is associated with brain diseases and neoplasms; and the gene *TNF* is associated with myocardial infarction. We found supporting literature for all these associations, which suggests that although genes are *not* annotated with the aforementioned diseases in our dataset, there may be evidence on the contrary.

## Supplementary Material

btz887_Supplementary_Data
