# Supplementary material for: Using deep learning to associate human genes with age-related diseases
Source: Bioinformatics. 2019 Dec 17;36(7):2202–8. doi: 10.1093/bioinformatics/btz887 (PMC7141856; doi:10.1093/bioinformatics/btz887)
Supplement: btz887_Supplementary_Data [file btz887_supplementary_data.zip › btz887-suppl_data/suppl.pdf]

Supplementary material

Table 1: List of negative genes with average positive class label probability greater than 0.5 across 30 randomised runs. The table shows the class label associated with the gene, the average, minimum and maximum probabilities across the 30 randomised runs.

Candidate gene	Avg. prob.	Min. prob.	Max. prob.
class_Disease.Immune (99% percentile of avg. prob.: 0.0257)			
APOE (apolipoprotein E)	0.1235	0.0315	0.2514
AGT (angiotensinogen)	0.1192	0.0307	0.2406
IL1B (interleukin 1 beta)	0.1036	0.0254	0.2142
LEP (leptin)	0.0983	0.0286	0.1934
APOA4 (apolipoprotein A4)	0.0981	0.0273	0.1975
class_Neoplasm.Breast (99% percentile of avg. prob.: 0.0669)			
TNF (tumor necrosis factor)	0.4119	0.1368	0.6384
AGT (angiotensinogen)	0.3923	0.1311	0.6027
IL1B (interleukin 1 beta)	0.3443	0.1130	0.5401
LEP (leptin)	0.3223	0.1113	0.4863
APOA4 (apolipoprotein A4)	0.3209	0.1081	0.4944
class_Heart.Coronary.Disease (99% percentile of avg. prob.: 0.0521)			
LEP (leptin)	0.2566	0.0436	0.4582
PTGS2 (prostaglandin-endoperoxide synthase 2)	0.2384	0.0424	0.4201
TGFB1 (transforming growth factor beta 1)	0.2336	0.0407	0.4089
IFNG (interferon gamma)	0.2193	0.0392	0.3889
PPARGC1A (PPARG coactivator 1 alpha)	0.1903	0.0340	0.3307
class_Disease.Nutrition (99% percentile of avg. prob.: 0.0698)			
IL1B (interleukin 1 beta)	0.4108	0.1478	0.6794
TGFB1 (transforming growth factor beta 1)	0.3492	0.1279	0.5670
AGTR2 (angiotensin II receptor type 2)	0.2533	0.1004	0.4096
EDN1 (endothelin 1)	0.2299	0.0900	0.3706
GSTP1 (glutathione S-transferase pi 1)	0.2247	0.0902	0.3606
class_Immune.Hypersensitivity (99% percentile of avg. prob.: 0.0299)			
APOE (apolipoprotein E)	0.1638	0.0750	0.2445
TNF (tumor necrosis factor)	0.1636	0.0738	0.2500
AGT (angiotensinogen)	0.1561	0.0706	0.2362
IL1B (interleukin 1 beta)	0.1365	0.0601	0.2045
LEP (leptin)	0.1294	0.0617	0.1973
class_Heart.Arteriosclerosis (99% percentile of avg. prob.: 0.0660)			
IL1B (interleukin 1 beta)	0.3489	0.1448	0.4838
TGFB1 (transforming growth factor beta 1)	0.2959	0.1240	0.4113
VEGFA (vascular endothelial growth factor A)	0.2878	0.1233	0.4006
IFNG (interferon gamma)	0.2796	0.1215	0.3942
APOA1 (apolipoprotein A1)	0.2780	0.1266	0.3860
class_Heart.Hypertension (99% percentile of avg. prob.: 0.0797)			
PTGS2 (prostaglandin-endoperoxide synthase 2)	0.3856	0.1380	0.5992
VEGFA (vascular endothelial growth factor A)	0.3677	0.1313	0.5777
IFNG (interferon gamma)	0.3573	0.1307	0.5494
APOA1 (apolipoprotein A1)	0.3551	0.1379	0.5391
PPARG (peroxisome proliferator activated receptor gamma)	0.3209	0.1163	0.4974
class_Disease.Muscle (99% percentile of avg. prob.: 0.0699)			
APOE (apolipoprotein E)	0.4223	0.1622	0.5975
AGT (angiotensinogen)	0.4016	0.1523	0.5668
LEP (leptin)	0.3312	0.1338	0.4625
APOA4 (apolipoprotein A4)	0.3300	0.1281	0.4664
PTGS2 (prostaglandin-endoperoxide synthase 2)	0.3064	0.1183	0.4346
class_Neoplasm.Stomach (99% percentile of avg. prob.: 0.0595)			
APOE (apolipoprotein E)	0.3425	0.1561	0.4799
AGT (angiotensinogen)	0.3262	0.1469	0.4634
LEP (leptin)	0.2676	0.1253	0.3843
APOA4 (apolipoprotein A4)	0.2673	0.1207	0.3759
IFNG (interferon gamma)	0.2292	0.1051	0.3263

Table 2: List of negative genes with average positive class label probability greater than 0.5 across 30 randomised runs. The table shows the class label associated with the gene, the average, minimum and maximum probabilities across the 30 randomised runs.

Candidate gene	Avg. prob.	Min. prob.	Max. prob.
class_Nutritional.Obesity (99% percentile of avg. prob.: 0.0866)			
IL1B (interleukin 1 beta)	0.4656	0.2013	0.6516
PTGS2 (prostaglandin-endoperoxide synthase 2)	0.4045	0.1817	0.5569
TGFB1 (transforming growth factor beta 1)	0.3967	0.1752	0.5445
VEGFA (vascular endothelial growth factor A)	0.3837	0.1714	0.5358
IFNG (interferon gamma)	0.3735	0.1705	0.5181
class_Muscle.Arthritis (99% percentile of avg. prob.: 0.0287)			
APOE (apolipoprotein E)	0.1427	0.0462	0.2833
AGT (angiotensinogen)	0.1366	0.0438	0.2712
LEP (leptin)	0.1132	0.0361	0.2267
APOA4 (apolipoprotein A4)	0.1125	0.0373	0.2241
PTGS2 (prostaglandin-endoperoxide synthase 2)	0.1052	0.0351	0.2092
class_Brain.Multiple.Sclerosis (99% percentile of avg. prob.: 0.0410)			
AGT (angiotensinogen)	0.2165	0.0977	0.3412
LEP (leptin)	0.1782	0.0847	0.2741
APOA4 (apolipoprotein A4)	0.1777	0.0816	0.2813
PTGS2 (prostaglandin-endoperoxide synthase 2)	0.1653	0.0762	0.2616
TGFB1 (transforming growth factor beta 1)	0.1617	0.0734	0.2551
class_Nutritional.Diabetes.Type2 (99% percentile of avg. prob.: 0.0897)			
IL1B (interleukin 1 beta)	0.4954	0.1451	0.8449
TGFB1 (transforming growth factor beta 1)	0.4211	0.1257	0.7044
AGTR2 (angiotensin II receptor type 2)	0.3030	0.0984	0.4955
EDN1 (endothelin 1)	0.2750	0.0876	0.4567
SLC6A4 (solute carrier family 6 member 4)	0.2719	0.0908	0.4438
class_Nutritional.Diabetes.Type1 (99% percentile of avg. prob.: 0.0650)			
APOE (apolipoprotein E)	0.4099	0.1965	0.6156
IL1B (interleukin 1 beta)	0.3437	0.1599	0.5234
LEP (leptin)	0.3207	0.1576	0.4683
APOA4 (apolipoprotein A4)	0.3200	0.1518	0.4805
PTGS2 (prostaglandin-endoperoxide synthase 2)	0.2964	0.1418	0.4457
class_Neoplasm.Colorectal (99% percentile of avg. prob.: 0.0783)			
TNF (tumor necrosis factor)	0.4710	0.1646	0.6528
APOE (apolipoprotein E)	0.4692	0.1674	0.6243
AGT (angiotensinogen)	0.4462	0.1561	0.5952
IL1B (interleukin 1 beta)	0.3928	0.1348	0.5336
LEP (leptin)	0.3679	0.1346	0.4999
class_Brain.Alzheimer (99% percentile of avg. prob.: 0.0321)			
AGT (angiotensinogen)	0.1613	0.0404	0.2882
LEP (leptin)	0.1328	0.0345	0.2348
APOA4 (apolipoprotein A4)	0.1324	0.0343	0.2362
PTGS2 (prostaglandin-endoperoxide synthase 2)	0.1243	0.0333	0.2220
TGFB1 (transforming growth factor beta 1)	0.1217	0.0317	0.2149
class_Neoplasm.Prostatic (99% percentile of avg. prob.: 0.0520)			
TNF (tumor necrosis factor)	0.3101	0.1008	0.5335
APOE (apolipoprotein E)	0.3098	0.1028	0.5307
AGT (angiotensinogen)	0.2951	0.0989	0.5039
IL1B (interleukin 1 beta)	0.2595	0.0851	0.4523
APOA4 (apolipoprotein A4)	0.2430	0.0822	0.4153
class_Disease.Brain (99% percentile of avg. prob.: 0.0942)			
AGT (angiotensinogen)	0.5764	0.2659	0.8631
LEP (leptin)	0.4762	0.2315	0.7186
APOA4 (apolipoprotein A4)	0.4737	0.2214	0.7014
PTGS2 (prostaglandin-endoperoxide synthase 2)	0.4393	0.2052	0.6542
TGFB1 (transforming growth factor beta 1)	0.4306	0.1977	0.6367

Table 3: List of negative genes with average positive class label probability greater than 0.5 across 30 randomised runs. The table shows the class label associated with the gene, the average, minimum and maximum probabilities across the 30 randomised runs.

Candidate gene	Avg. prob.	Min. prob.	Max. prob.
class_Neoplasm.Lung (99% percentile of avg. prob.: 0.0724)			
TNF (tumor necrosis factor)	0.4471	0.1731	0.6011
APOE (apolipoprotein E)	0.4469	0.1738	0.5862
AGT (angiotensinogen)	0.4261	0.1660	0.5674
IL1B (interleukin 1 beta)	0.3735	0.1416	0.4963
APOA4 (apolipoprotein A4)	0.3489	0.1370	0.4600
class_Muscle.Osteoporosis (99% percentile of avg. prob.: 0.0297)			
APOE (apolipoprotein E)	0.1632	0.0762	0.2251
TNF (tumor necrosis factor)	0.1629	0.0748	0.2254
AGT (angiotensinogen)	0.1549	0.0708	0.2130
LEP (leptin)	0.1276	0.0617	0.1706
APOA4 (apolipoprotein A4)	0.1274	0.0590	0.1754
class_Neoplasm.Adenocarcinoma (99% percentile of avg. prob.: 0.0469)			
TNF (tumor necrosis factor)	0.2677	0.1113	0.4094
APOE (apolipoprotein E)	0.2671	0.1102	0.3975
AGT (angiotensinogen)	0.2554	0.1020	0.3791
LEP (leptin)	0.2083	0.0891	0.3130
APOA4 (apolipoprotein A4)	0.2076	0.0865	0.3099
class_Disease.Neoplasm (99% percentile of avg. prob.: 0.0784)			
AGT (angiotensinogen)	0.5128	0.1728	0.8357
APOA4 (apolipoprotein A4)	0.4228	0.1439	0.6891
APOA1 (apolipoprotein A1)	0.3610	0.1338	0.5780
ADIPOQ (adiponectin, C1Q and collagen domain containing)	0.3507	0.1243	0.5686
APOB (apolipoprotein B)	0.2856	0.1036	0.4588
class_Disease.Heart (99% percentile of avg. prob.: 0.0797)			
IFNG (interferon gamma)	0.4299	0.0940	0.8144
CD36 (CD36 molecule)	0.3342	0.0779	0.6220
IL10 (interleukin 10)	0.2931	0.0688	0.5426
GSTP1 (glutathione S-transferase pi 1)	0.2922	0.0672	0.5514
APOC2 (apolipoprotein C2)	0.2872	0.0672	0.5458
class_Heart.Myocardial.Infarction (99% percentile of avg. prob.: 0.0808)			
TNF (tumor necrosis factor)	0.5283	0.1279	0.8312
LEP (leptin)	0.4138	0.1050	0.6374
IFNG (interferon gamma)	0.3532	0.0888	0.5545
PPARGC1A (PPARG coactivator 1 alpha)	0.3050	0.0734	0.4603
CYP1A1 (cytochrome P450 family 1 subfamily A member 1)	0.2844	0.0736	0.4284
class_Brain.Parkinson (99% percentile of avg. prob.: 0.0423)			
TNF (tumor necrosis factor)	0.2455	0.1140	0.3499
AGT (angiotensinogen)	0.2321	0.1071	0.3311
LEP (leptin)	0.1920	0.0943	0.2783
APOA4 (apolipoprotein A4)	0.1908	0.0903	0.2707
PTGS2 (prostaglandin-endoperoxide synthase 2)	0.1769	0.0831	0.2494
class_Disease (99% percentile of avg. prob.: 0.0493)			
APOA2 (apolipoprotein A2)	0.2488	0.0834	0.4319
ABCG1 (ATP binding cassette subfamily G member 1)	0.1679	0.0615	0.2976
SERPINF2 (serpin family F member 2)	0.1421	0.0541	0.2421
APOH (apolipoprotein H)	0.1312	0.0503	0.2080
CYP4F12 (cytochrome P450 family 4 subfamily F member 12)	0.1270	0.0503	0.2136
class_Respiratory.Asthma (99% percentile of avg. prob.: 0.0716)			
APOE (apolipoprotein E)	0.4295	0.1759	0.6403
AGT (angiotensinogen)	0.4080	0.1647	0.6112
LEP (leptin)	0.3373	0.1429	0.5137
APOA4 (apolipoprotein A4)	0.3352	0.1376	0.5073
PTGS2 (prostaglandin-endoperoxide synthase 2)	0.3113	0.1284	0.4705

Table 4: List of negatively labelled candidate genes with at least 9 positive neighbours annotated with the label 'Associated with Type 2 Diabetes' appearing in at least 20 out of the 30 randomised runs of the Modular DNN. Each sub-table shows in its heading the name of the candidate gene and the average, minimum and maximum positive class label probabilities across the 30 randomised runs. Next, we show the list of positive neighbours and the list of its negative neighbours (if any) of the candidate gene. The sub-tables also show the number of times the gene was in the Nearest Neighbour (NN) list of the candidate gene.

Class label: Associated with Type 2 Diabetes

Candidate gene: **AGTR2 (angiotensin II receptor type 2)**. Found in 29 out of 30 randomised runs.
Avg. prob.: 0.3030 / Min. prob.: 0.0984 / Max. prob.: 0.4955

Times in NN list	Positive neighbouring genes
29	ADRB2 (adrenoceptor beta 2)
29	CD36 (CD36 molecule)
29	INS (insulin)
29	TLR4 (toll like receptor 4)
29	NOS3 (nitric oxide synthase 3)
29	INSR (insulin receptor)
29	APOB (apolipoprotein B)
28	CYP1A1 (cytochrome P450 family 1 subfamily A member 1)
23	ADRB3 (adrenoceptor beta 3)
9	APOA5 (apolipoprotein A5)
4	PPARGC1A (PPARG coactivator 1 alpha)
3	CCL2 (C-C motif chemokine ligand 2)

Times in NN list	Negative neighbouring genes
13	EDN1 (endothelin 1)
4	SLC6A4 (solute carrier family 6 member 4)
2	IL10 (interleukin 10)
1	GSTP1 (glutathione S-transferase pi 1)

Table 5: List of negatively labelled candidate genes with at least 9 positive neighbours annotated with 'Associated with Heart Diseases' appearing in at least 20 out of the 30 randomised runs of the Modular DNN. Each sub-table shows in its heading the name of the candidate gene and the average, minimum and maximum positive class label probabilities across the 30 randomised runs. Next, we show the list of positive neighbours and the list of its negative neighbours (if any) of the candidate gene. The sub-tables also show the number of times the gene was in the Nearest Neighbour (NN) list of the candidate gene.

Class label: Associated with Heart Diseases

Candidate gene: **CD36 (CD36 molecule)**. Found in 30 out of 30 randomised runs.
Avg. prob.: 0.3342 / Min. prob.: 0.0779 / Max. prob.: 0.6220

Times in NN list	Positive neighbouring genes
30	APOB (apolipoprotein B)
30	CYP1A1 (cytochrome P450 family 1 subfamily A member 1)
30	INS (insulin)
30	AGTR2 (angiotensin II receptor type 2)
30	INSR (insulin receptor)
30	ADRB2 (adrenoceptor beta 2)
29	TLR4 (toll like receptor 4)
29	NOS3 (nitric oxide synthase 3)
24	ADRB3 (adrenoceptor beta 3)
12	EDN1 (endothelin 1)
8	PPARGC1A (PPARG coactivator 1 alpha)
7	SLC6A4 (solute carrier family 6 member 4)
6	APOA5 (apolipoprotein A5)
2	PPARG (peroxisome proliferator activated receptor gamma)
1	CCL2 (C-C motif chemokine ligand 2)
**Times in NN list**	**Negative neighbouring genes**
1	GSTP1 (glutathione S-transferase pi 1)
1	IL10 (interleukin 10)

Candidate gene: **IFNG (interferon gamma)**. Found in 30 out of 30 randomised runs.
Avg. prob.: 0.4299 / Min. prob.: 0.0940 / Max. prob.: 0.8144

Times in NN list	Positive neighbouring genes
30	APOA1 (apolipoprotein A1)
30	ADIPOQ (adiponectin, C1Q and collagen domain containing)
30	PPARGC1A (PPARG coactivator 1 alpha)
30	PTGS2 (prostaglandin-endoperoxide synthase 2)
30	VEGFA (vascular endothelial growth factor A)
30	PPARG (peroxisome proliferator activated receptor gamma)
30	TGFB1 (transforming growth factor beta 1)
26	LEP (leptin)
25	APOA4 (apolipoprotein A4)
16	CYP1A1 (cytochrome P450 family 1 subfamily A member 1)
16	INS (insulin)
4	APOB (apolipoprotein B)
2	IL1B (interleukin 1 beta)
1	AGTR2 (angiotensin II receptor type 2)

Table 6: List of negatively labelled candidate genes with at least 9 positive neighbours annotated with 'Associated with Myocardial Infarction' appearing in at least 20 out of the 30 randomised runs of the Modular DNN. The sub-table shows in its heading the name of the candidate gene, followed by the list of its positive neighbours and its single negative neighbour. The sub-table also shows the number of times the gene was in the Nearest Neighbour (NN) list of the candidate gene.

Class label: Associated with Myocardial Infarction

Candidate gene: **LEP (leptin)**. Found in 27 out of 30 randomised runs.
Avg. prob.: 0.4138 / Min. prob.: 0.1050 / Max. prob.: 0.6374

Times in NN list	Positive neighbouring genes
27	IL1B (interleukin 1 beta)
27	APOA1 (apolipoprotein A1)
27	ADIPOQ (adiponectin, C1Q and collagen domain containing)
27	APOA4 (apolipoprotein A4)
27	AGT (angiotensinogen)
27	PTGS2 (prostaglandin-endoperoxide synthase 2)
27	TGFB1 (transforming growth factor beta 1)
27	VEGFA (vascular endothelial growth factor A)
22	PPARG (peroxisome proliferator activated receptor gamma)
5	APOE (apolipoprotein E)
**Times in NN list**	**Negative neighbouring gene**
27	IFNG (interferon gamma)
